# An intricate relationship: stress markers and associative memory in a laboratory experiment in older adults

**DOI:** 10.3389/fnagi.2025.1666566

**Published:** 2025-10-29

**Authors:** Luisa Knopf, Gregor Domes, Siri-Maria Kamp

**Affiliations:** ^1^Department of Neurocognitive Psychology, University of Trier, Trier, Germany; ^2^Department of Biological and Clinical Psychology, Institute for Cognitive & Affective Neuroscience (ICAN), University of Trier, Trier, Germany; ^3^Department of Neurocognitive Psychology, Institute for Cognitive & Affective Neuroscience (ICAN), University of Trier, Trier, Germany

**Keywords:** cognitive aging, memory, cortisol, affective response, neuroendocrine response

## Abstract

**Introduction:**

Researchers working in the field of cognitive aging frequently encounter highly motivated yet nervous older participants during data collection in the laboratory. Such anecdotal experiences raise the question of whether the affective or physiological response of older participants to psychological laboratory experiments differs to that of young adults, who might be less motivated but also less nervous, as they may be more used to the environment and to learning and memory tests.

**Methods:**

In the present study, we collected saliva samples and subjective affective ratings during an EEG experiment on memory, and at home, in young and older adults, while also taking into account sex effects.

**Results:**

There was no significant interaction involving time point (laboratory vs. at home) and age group. However, across both time points older males showed significantly higher cortisol-levels than older females, while there was no difference for younger males and females. The trajectories in cortisol levels throughout the session, especially around the memory task, differed by age: While there was a decrease in cortisol levels for younger adults from before to after the memory task, we did not observe such a decrease in older participants. There were few age differences in alpha-amylase or negative affect. However, older adults showed higher ratings of positive affect than younger participants. Importantly, lower cortisol levels before the memory task were associated with higher associative memory performance for older adults.

**Discussion:**

Affective reactions to psychological laboratory tasks may hence be an important factor to consider in psychological experiments in the field of cognitive aging.

## Introduction

In laboratory-based psychological studies with older participants, research groups including ours are often confronted with participants' insecurities concerning the laboratory setting and the psychological (or neural) measures being collected. Also, older participants often express concerns about their cognition declining (e.g., Jessen et al., 2014). Although explanations and interactions with the research team may attenuate such effects, older adults may nevertheless differ in their affective response in psychological laboratory studies from young adults, who are more familiar with the environment and with memory testing. This, in turn, may affect the dependent variables of interest, such as memory performance. It is hence important to investigate age differences in affective responses within laboratory studies and their effects on performance.

Although there is little research examining the effect of the laboratory setting on stress parameters in older adults, in one study, older participants had significantly increased cortisol levels at the beginning of the laboratory session, compared to younger adults. However, these differences were mitigated by a prior group session where the older adults got to know the setting and staff members before the experimental session (Lupien et al., 2007). Examining the effect of the test setting on memory performance, Schlemmer and Desrichard (2018) showed that testing older adults in a medical environment enhanced performance in participants with higher memory self-efficacy, whereas those with lower memory self-efficacy performed worse. Turning to a reaction to task characteristics, a slight change in task instructions can have a differential effect on memory in young and older adults: Rahhal et al. (2001) observed differences in performance when the instructions highlighted the memory component of a task, but not when more neutral instructions were used. Further studies have shown that activating age-based stereotypes can reduce cognitive performance in various tasks (for reviews, see Barber, 2020; Dionigi, 2015; also see Lamont et al., 2015 for a meta-analysis). Taken together, older adults may show elevated affective and stress markers, as well as modulated memory performance due to the test setting, as well as characteristics and framing of the task itself. To sharpen interpretations from laboratory research in the field of cognitive aging, it is hence important to systematically examine whether older adults differ in physiological and psychological stress markers in a laboratory task, and their association with memory performance.

To address this issue, in the present study, we collected different physiological and psychological stress markers from young and older adults during a laboratory-based EEG-experiment including a difficult associative memory task. The three main research questions were: (1) Does the laboratory context enhance stress markers in older adults (i.e., at the beginning of the session)?, (2) Does a difficult memory task modulate trajectory of stress markers during the session in older adults?, and (3) Are physiological stress markers in a laboratory experiment associated with associative memory performance in older adults?

### Stress and cortisol effects on memory performance

Memory processes have long been known to be sensitive to stress, whereas factors like stressor timing and relevance of the material to the stressor (for reviews, see Lupien et al., 2007; Shields et al., 2017), as well as and time of the day (Het et al., 2005) play a role. Notably, older adults have a strong difficulty encoding and retrieving associations between different information units (“items”), whereas memory for single items is less impaired (Old and Naveh-Benjamin, 2008). When examining how stress markers in a laboratory experiment affect memory in older adults, it is hence important consider how stress affects associative (vs. item) memory in general.

Item and associative memory may be affected differently by stress, because item memory relies on a combination of recollection and familiarity, but associative memory depends primarily on recollection (McCullough et al., 2015; Yonelinas, 1997). Findings regarding potentially differential effects on associative and item memory have been complex and heterogeneous in the literature. Exploring the impact of cortisol on memory performance, Sherman et al. (2023) reported that pre-encoding hydrocortisone administration increases the functional connectivity among subregions of the hippocampus, which was linked to increased memory for emotional associations. In another study, associative memory for high-arousal word-picture pairs increased after pre-encoding stress, while item memory benefited from post-encoding stress (Goldfarb et al., 2019). Further, a pre-enconding stress-induced increase in memory performance has been reported for item, but not associative recognition (Kamp et al., 2019), while a conditioned evaluative response (a form of learned association) was abolished by pre-retrieval stress (Halbeisen et al., 2020). Taken together, some research hints toward a particular sensitivity of associative memory to stress, but the association seems to be complex and depend on factors like stressor timing.

If the laboratory setting elicits a stress response in older adults, higher cortisol levels should be observed in older compared to young adults at the beginning of the session. In this case, the laboratory context could be considered a pre-encoding stressor. If the task itself elicits a stress response, the effect on memory performance may be more intricate as the stressor persists during encoding, consolidation, and retrieval. The present study hence aims to contribute to a better understanding of how these factors may influence memory performance in older adults.

### Effects of age on the physiological stress markers

Another important finding is that the stress response itself differs by age. Indeed, older adults seem to show an increased response to (pharmacological or psychological) stress (Gotthardt et al., 1995; Kudielka et al., 2004; Otte et al., 2005), with potentially higher age effects on cortisol levels in women than men (Otte et al., 2005). Together with other findings of sex-specific stress effects (Almela et al., 2011; Antypa et al., 2022), the latter finding highlights the importance of considering sex when studying age effects on stress responses.

Importantly, the interaction of stress with cognition may also differ by age: For example, Gutchess et al. (2019) reported that higher cortisol levels were associated with a reduced trade-off between enhanced memory for emotional information at the expense of background information in older vs. young adults.

Overall, aging appears to modulate the physiological stress response and its effects on memory, with sex potentially playing a role. The complex and heterogeneous patterns of prior findings highlights the importance of the present study.

### The present study

In the present study, we collected different stress measures (salivary cortisol, alpha-amylase, subjective affect) at several time points throughout the first of two experimental sessions during a larger study, of which several subsets of the data set have been previously reported. The session consisted of the preparation for an EEG recording, followed by a difficult memory task (Kamp, 2020a), a simple reaction time task (Kamp, 2020b), a working memory task, as well as a short resting EEG (Kamp et al., 2021; see also Kamp et al., 2022 and Kamp et al., 2023; for procedures and results from the second session, in which no stress markers were collected).

Additionally, for an assessment of baseline cortisol, participants were asked to collect two saliva samples at home during the same time of day as the laboratory session.

First, we hypothesized that if the laboratory context acts as a stressor, the older participants' salivary cortisol, alpha-amylase and negative affect levels would be significantly higher at the start of the session than the younger adults' cortisol levels. Secondly, we investigated the influence of the difficult memory task on the stress level. If the task elicited a stress response in older adults, the trajectory of the stress markers throughout the session should differ and especially diverge between the age groups during the task. Finally, we examined whether physiological and psychological markers of stress correlated with associative memory performance in the older adults. Sex effects were examined in each analysis exploratorily.

## Method

### Participants

Fifty-four older and 30 young adults participated in the study in exchange for a compensation of 10 Euros per hour or partial course credit. Two older adults were excluded from further analysis as they reported a history of a stroke, resulting in a data set including 52 older (*M* = 70.17 years, *SD* = 4.66) and 30 young (*M* = 24.73 years, *SD* = 3.66) participants. 26 older and 19 younger participants were female, and the remainder were male. For analyses including the “at-home” measures, 30 young and 49 older adults were included, as 3 older participants failed to collect saliva samples at home. Neuropsychological test results of the older group have been previously reported (e.g., Kamp, 2020a) and no participants showed signs of clinically relevant cognitive decline.

The sample size was planned a priori to address the main aims of the original study (Kamp, 2020a). Given the resource-intensive nature of the study, the sample sizes reflect a balance between feasibility and sufficient statistical power for the intended analyses. To examine the feasibility of the collected sample for the present research question, we conducted *post-hoc* sensitivity analyses with G^*^Power 3.1 to determine the minimum effect size detectable with the present sample.

Given our total sample size of 82, an alpha level of 0.05 and a desired power of 0.80 we are able to detect a small to medium effect size of *f* = 15.6 for the baseline comparisons (at home vs. in the laboratory in young vs. older adults, interaction effect), and *f* = 13.0 for the trajectory analyses (four measurements throughout the session in young vs. older adults, interaction effect). Excluding outliers in different analyses increased the detectible effect size somewhat. For the correlational analyses only conducted in older adults (*n* = 49 after the exclusion of outliers), we have sufficient power to detect correlations of *r* = 0.24.

### Procedure

**Figure 1:**
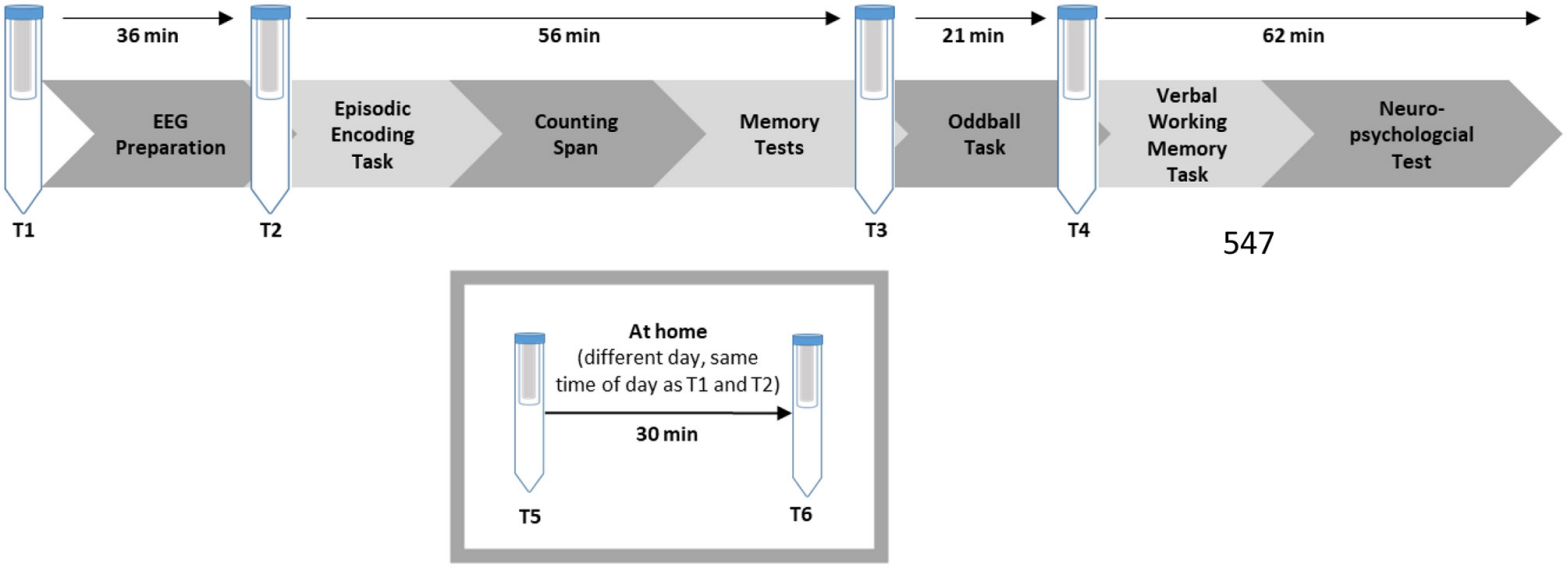
Overview of the study procedure.

The data for the present study was collected as part of a larger study, which will be described briefly. For an overview refer to Figure 1. Older participants were recruited via a newspaper advertisement. First, all older participants filled out a packet of paper-based questionnaires at home, which also contained an invitation letter including the instruction to refrain from smoking, eating, or drinking sugary drinks 30 min ahead of the session, and not to drink alcohol the day before. The young adults received the same instruction digitally upon their registration for the study.

**Figure 2:**
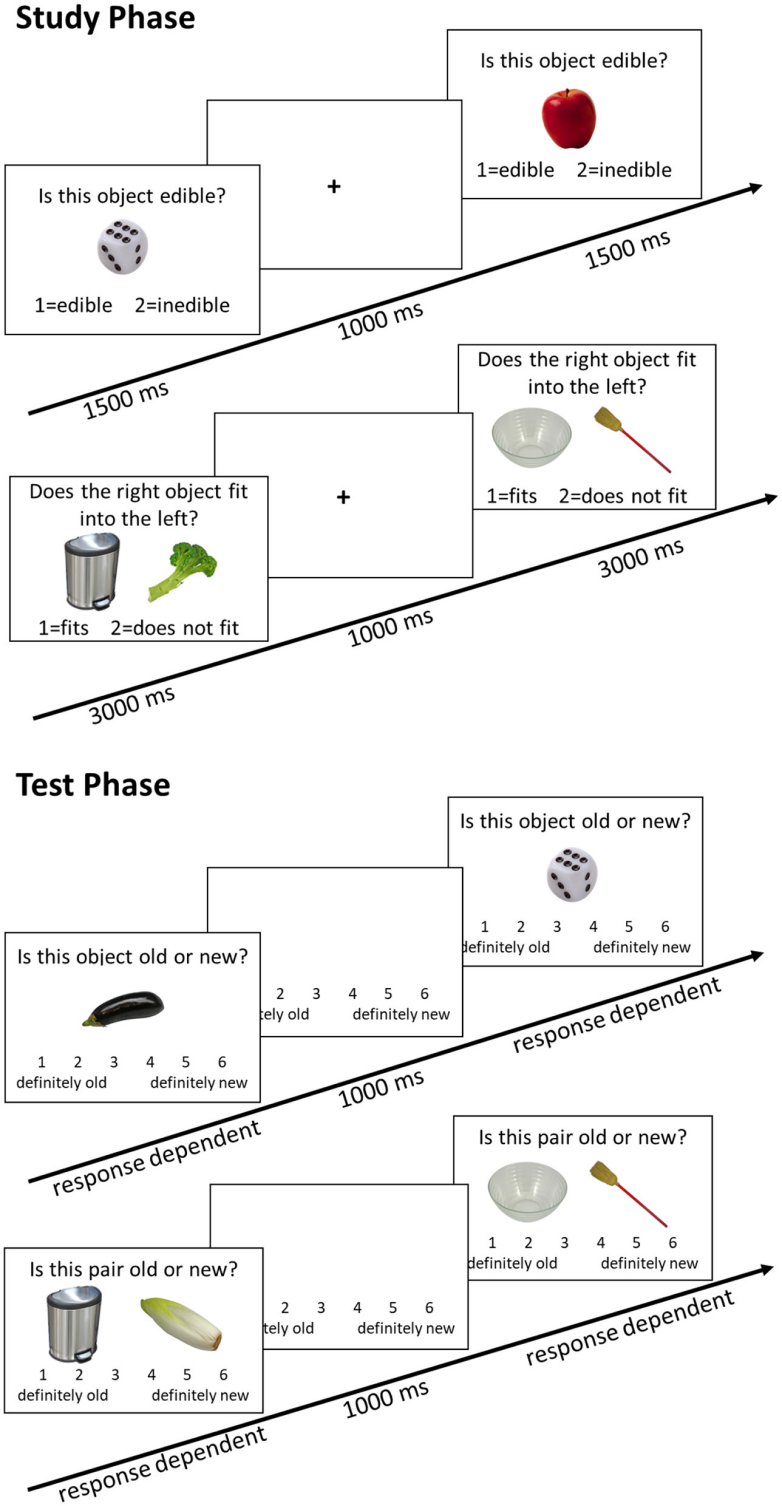
Memory task trial structure. In both the study phase and the test phase, the first sequence depicts the item memory task, and the second sequence depicts the associative memory task. Only performance in the associative memory task was analyzed in the present study.

Upon arrival in the laboratory, all participants signed an informed consent form, and the first saliva sample was collected. Afterward, the EEG preparation took place, which took maximally 45 min. During the EEG preparation, the participants filled out a questionnaire concerning demographic data and interacted with the experimenter. Afterwards, the second saliva sample was taken. Next, an episodic memory task followed (the behavioral and EEG results were published in Kamp, 2020a). To implement earlier findings that task instructions can alter performance (Rahhal et al., 2001; Sindi et al., 2013), we refrained from emphasizing high performance and included practice trials to allow participants to familiarize themselves with the task (for an English translation of the exact instructions, see Supplementary materials). During the learning phase, participants were first presented with pictures of 60 objects and judged whether the object presented was edible or not. After the item encoding phase, participants were presented with 92 object pairs and asked if one could fit into the other. After a 15-min break during which the participants completed a demanding computerized working memory task, an item and associative memory test followed. In the item memory test, all 60 items from the learning phase and 32 novel pictures were randomly presented. The participants judged whether it was an “old” or a “new” item on a six-point Likert scale. During the associative memory test, 60 previously learned pairs and 32 newly combined pairs were presented. The participants again judged whether the combination was “old” or “new” on a six-point Likert scale. The task lasted a total of about 45 min (see Figure 2 or Kamp, 2020a for more details). After completing the episodic memory task, a third saliva sample was taken. The participants could then take a self-paced break and then continue with a simple reaction time task (oddball task), which lasted about 20 min (see Kamp, 2020b; Kamp et al., 2023, for details). After completing the oddball task, a fourth saliva sample was taken. An unrelated verbal working memory task and several neuropsychological tests followed. The entire session lasted about 3.5–4 h. At the end of the session, the participants were given two Salivettes to collect saliva samples at home, as well as two PANAS questionnaires. The participants were instructed to collect the saliva sample and fill out the PANAS, on an ordinary day at the same time as their laboratory session started, and 30 min later. They were also asked to document the exact time on a provided form and return the samples to our laboratory via mail.

### Sampling and analysis of saliva cortisol and alpha-amylase

For the saliva samples, Cortisol-Salivettes (Sarstedt, Nümbrecht-Rommelsdorf, Germany) were used at four times during the laboratory session and twice at home: (T1) at the beginning of the session, (T2) after EEG preparation, (T3) after the episodic memory task, (T4) after the oddball task, (T5) at home at the same time as the start of the session, (T6) at home 30 min later.

In order to determine the cortisol concentration in the saliva sample we used a time-resolved fluorescence immunoassay (Dressendörfer et al., 1992). The intra-assay coefficient of variation was between 4.0% and 6.7%, and the corresponding inter-assay coefficients of variation were between 7.1% and 9.0%.

The alpha-amylase Assay is based on an enzymatic action of the alpha-amylase (Lorentz et al., 1999; Winn-Deen et al., 1988). The intra-assay coefficient of variation was between 2.8% and 6.3%, and the corresponding inter-assay coefficients of variation were between 5.5% and 7.6%.

### Subjective negative (NA) and positive affect (PA)

To assess participants' affective state the German version of the Positive and Negative Affective Schedule (PANAS; Krohne et al., 1996) was used. The questionnaire was filled out at the same time as saliva samples were taken.

### Statistical analysis

We statistically analyzed all dependent variables in mixed ANCOVAs with the within-subject factor time point and the between subject factors age group (young vs. old) and sex (male vs. female), using IBM SPSS 28 software. Greenhouse-Geisser correction was applied where necessary. The start time of the experimental session was included in all ANCOVAs as a covariate to account for the diurnal cycle of cortisol (O'Byrne et al., 2021).^1^ We report partial eta squared (η^2^_*p*_) as a measure of effect size. Significant main effects of factors with more than one level and interactions were followed up by lower level ANCOVAs or Fishers LSD.

Since the distribution of cortisol and alpha-amylase were positively skewed, in a Supplementary analysis, we log(ln)-transformed these data and repeated our ANOVA analyses. To allow for a direct interpretation of the physiological values and since ANOVA is considered robust against violations of statistical assumptions (Blanca Mena et al., 2017; Rayarao, 2025), we report the analysis of the raw cortisol and amylase data in our main article and refer to the Supplementary materials for analyses of the log-transformed data. Importantly, the main findings are comparable in both analyses, and our main conclusions do not differ regardless of which set of analyses is considered.

A bivariate correlation analysis was conducted to evaluate the relationship between stress response and associative memory performance. We calculated Spearman's Rho between all pre- and post-task stress measures with associative memory performance, measured as PR-score (hit rate minus false alarm rate in the associative memory task). For 8 correlation coefficients (2 time points, 4 stress measures each), a Bonferroni-correction suggests that a correlation coefficient is considered statistically significant if *p* < 0.00625.

Outliers in physiological and affective measures were detected before statistical analysis and were excluded from all analysis if they exceeded the expected range based on the interquartile range (IQR) criteria. Values outside the 3rd quartile + 3^*^interquartile range or 1st quartile – 3^*^interquartile range were considered outliers. Outliers exclusion was measure-specific.

## Results

For all means and standard deviations refer to Table 1.

**Table 1:** Means and standard deviation for all variables.

		**Young adults**						**Older adults**					
		**Male (*****N*** = **11)**		**Female (*****N*** = **19)**		**Total (*****N*** = **30)**		**Male (*****N*** = **26)**		**Female (*****N*** = **26)**		**Total (*****N*** = **52)**	
		**M**	**SD**	**M**	**SD**	**M**	**SD**	**M**	**SD**	**M**	**SD**	**M**	**SD**
Cortisol (nmol/l)	T1	5.54	4.70	5.53	4.51	5.54	4.50	10.18	6.91	5.18	3.25	7.68	5.91
	T2	5.60	3.77	5.41	6.07	5.48	5.27	6.88	3.75	4.48	2.97	5.68	3.56
	T3	5.30	3.88	3.57	2.27	4.21	3.02	6.81	3.64	4.69	2.92	5.75	3.44
	T4	4.51	3.43	3.20	1.89	3.68	2.58	5.50	2.30	3.81	1.92	4.65	2.27
	T5	4.76	3.03	6.77	5.67	6.03	4.91	8.27	5.39	6.33	4.55	7.36	5.06
	T6	5.48	4.10	6.71	5.70	6.25	5.13	8.24	6.03	5.49	3.02	6.95	5.01
Alpha-amylase (U/ml)	T1	73.45	49.49	124.51	76.84	105.79	71.67	116.82	74.01	165.49	176.28	141.15	136.09
	T2	110.48	52.94	195.68	130.75	164.44	115.42	158.32	109.36	199.68	190.21	179.00	155.03
	T3	114.89	45.14	178.66	136.93	155.28	115.40	191.90	127.50	226.59	224.36	209.24	181.52
	T4	121.63	48.07	173.52	134.87	154.49	112.85	197.55	124.98	210.49	201.94	204.02	166.41
	T5	101.72	49.25	125.20	122.85	116.59	101.67	124.71	103.20	120.56	82.37	122.76	93.07
	T6	91.92	46.68	133.70	106.65	118.38	90.72	121.10	110.86	124.00	66.00	122.46	91.65
Positive affect	T1	2.58	0.53	2.86	0.66	2.76	0.62	3.33	0.37	3.19	0.71	3.26	0.57
	T2	2.49	0.66	2.67	0.68	2.60	0.67	3.32	0.50	3.18	0.82	3.25	0.68
	T3	2.58	0.78	2.59	0.70	2.59	0.72	3.32	0.56	3.05	0.74	3.19	0.67
	T4	1.97	0.70	2.15	0.74	2.09	0.72	3.37	0.59	3.13	0.85	3.25	0.73
	T5	2.60	0.64	2.42	0.75	2.49	0.70	2.84	0.69	3.01	0.89	2.92	0.79
	T6	2.73	0.75	2.45	0.67	2.55	0.70	2.91	0.51	3.21	0.76	3.05	0.65
Negative affect	T1	1.11	0.10	1.18	0.20	1.15	0.17	1.14	0.16	1.12	0.12	1.13	0.14
	T2	1.05	0.08	1.07	0.11	1.07	0.10	1.07	0.09	1.09	0.17	1.08	0.13
	T3	1.14	0.20	1.08	0.16	1.10	0.18	1.13	0.15	1.22	0.35	1.17	0.27
	T4	1.09	0.18	1.11	0.15	1.10	0.16	1.12	0.14	1.17	0.35	1.14	0.27
	T5	1.34	0.41	1.15	0.19	1.22	0.30	1.09	0.15	1.07	0.13	1.08	0.14
	T6	1.29	0.35	1.13	0.26	1.18	0.30	1.10	0.24	1.13	0.38	1.11	0.31
Item memory performance (PR)		0.82	0.12	0.78	0.13	0.80	0.13	0.67	0.19	0.59	0.22	0.63	0.21
Associative memory performance (PR)		0.39	0.21	0.32	0.16	0.35	0.18	0.15	0.12	0.18	0.08	0.17	0.10
Start time (hh:mm)		12:26	3:17	12:23	3:04	12:24	3:06	10:33	3:21	11:08	2:29	10:51	2:56

The PR-Score is calculated by subtracting the false alarm rate from the hit rate. One older male participant did not complete the questionnaire at home, resulting in a sample size of N = 25 for positive and negative affect. In addition, three older female participants did not complete the salivary sampling and questionnaire at home; therefore, the sample size for T5 and T6 is N = 23. To improve readability, related rows have been highlighted in color.

### Cortisol

One young female was excluded from all analysis of cortisol as an outlier.

#### Baseline cortisol

A 2 (time point: T1 vs. T5) × 2 (age group) × 2 (sex) mixed ANCOVA revealed a significant effect of the covariate, *F*_(1, 73)_ = 80.08, *p* < 0.001, η^2^_*p*_ = 0.52, and a significant interaction of time point and sex, *F*_(1, 73)_ = 6.14, *p* = 0.016, η^2^_*p*_ = 0.08. For males, cortisol levels were significantly higher at the start of the session than for females, *p* = 0.004, *M*_*Diff*_ = 2.66, 95%-*CI* [0.88, 4.44], while there were no significant sex differences for the samples taken at home, *p* = 0.750, *M*_*Diff*_ = −0.31, 95%-*CI* [−2.25, 1.63]. This was driven by the fact that females had a lower cortisol level at the beginning of the session than at home, *p* = 0.044, *M*_*Diff*_ = −1.62, 95%-*CI* [−3.20, −0.04], while males did not differ in their cortisol levels between the session and at home, *p* > 0.138. Furthermore, a significant age group × sex interaction, *F*_(1, 73)_ = 7.76, *p* = 0.007, η^2^_*p*_ = 0.10, revealed that, overall, older males had significantly higher cortisol levels than older females, *p* < 0.001, *M*_*Diff*_ = 3.17, 95%-*CI* [1.45, 4.88], while there was no significant difference between younger males and females, *p* = 0.479, *M*_*Diff*_ = −0.82, 95%-*CI* [−3.10, 1.47]. There were no other main or interaction effects (all *p*-values > 0.43).

#### Cortisol levels throughout the session

A 4 (time point: T1–T4) × 2 (age group) × 2 (sex) mixed ANCOVA, revealed a significant effect of the covariate, *F*_(1, 76)_ = 51.02, *p* < 0.001, η^2^_*p*_ = 0.40, and of sex, *F*_(1, 76)_ = 11.20, *p* = 0.001, η^2^_*p*_ = 0.13, such that males had higher cortisol levels than females. There was a main effect of time point, *F*_(1.64, 124.34)_ = 20.28, *p* < 0.001, η^2^_*p*_ = 0.21, and an interaction of time point with the covariate, *F*_(1.64, 124.34)_ = 13.61, *p* < 0.001, η^2^_*p*_ = 0.15. Crucially, a three-way interaction of time point, age group, and sex was observed, *F*_(1.64, 124.34)_ = 3.79, *p* = 0.03, η^2^_*p*_ = 0.05. To follow up on the three-way interaction, separate 4 (time point) × 2 (sex) ANCOVAs were calculated for the young and older adults.

#### Young adults

**Figure 3:**
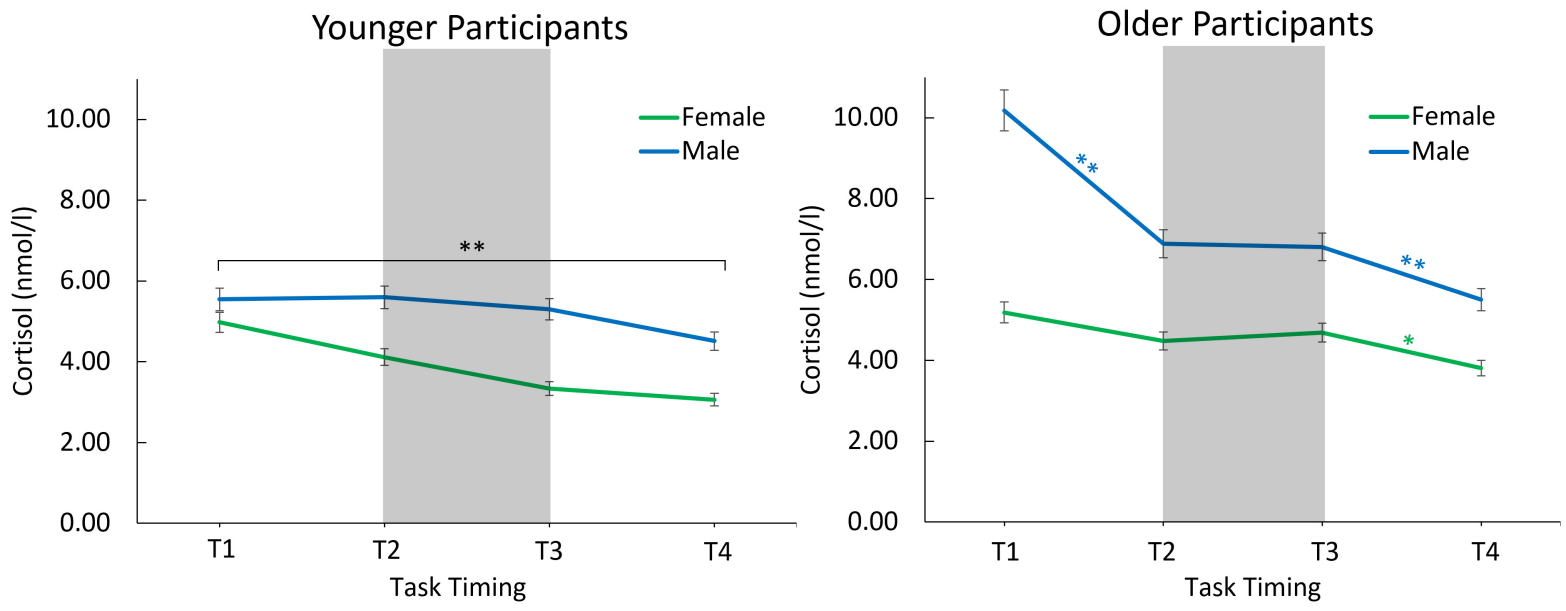
Estimated mean cortisol levels with consideration of the covariate start time of older and younger males and females throughout the session. The error bars indicate the 95% confidence interval. *Indicates significance <0.05, **indicates significance <0.01. Between T1 and T2, EEG preparation took place. Between T2 and T3, participants completed a memory task, and between T3 and T4, an oddball task was performed.

For the young adults, there was a significant effect of the covariate, *F*_(1, 26)_ = 40.16, *p* < 0.001, η^2^_*p*_ = 0.61, and of time point, *F*_(1.47, 38.11)_ = 13.72, *p* < 0.001, η^2^_*p*_ = 0.35. Regarding the main effect of time point, both the linear, *F*_(1, 26)_ = 19.19, *p* < 0.001, η^2^_*p*_ = 0.43, and the quadratic trend were significant, *F*_(1, 26)_ = 4.75, *p* = 0.04, η^2^_*p*_ = 0.15. There was no main or interaction effect involving the factor sex. Cortisol levels gradually declined throughout the session (Figure 3).

#### Older adults

For the older adults, aside from an effect of the covariate, *F*_(1, 49)_ = 22.23, *p* < 0.001, η^2^_*p*_ = 0.31, there were main effects of sex, *F*_(1, 49)_ = 11.94, *p* =0.001, η^2^_*p*_ = 0.20, and time point, *F*_(1.64, 80.37)_ = 11.54, *p* < 0.001, η^2^_*p*_ = 0.19, as well as a significant interaction effect of the covariate and time point, *F*_(1.64, 80.37)_ = 6.39, *p* = 0.005, η^2^_*p*_ = 0.12. Most importantly, sex interacted with time point, *F*_(1.64, 80.37)_ = 4.09, *p* = 0.027, η^2^_*p*_ = 0.08, suggesting different trajectories in cortisol levels for men and women (Figure 3).

A one-way ANCOVA with the factor time point (T1–T4) for the men revealed a significant effect of the covariate, *F*_(1, 24)_ = 17.64, *p* < 0.001, η^2^_*p*_ = 0.42, a main effect of time point, *F*_(1.52, 36.52)_ = 13.50, *p* < 0.001, η^2^_*p*_ = 0.36, as well as an interaction of the covariate with time point, *F*_(1.52, 36.52)_ = 7.31, *p* = 0.004, η^2^_*p*_ = 0.23. Significant decreases in cortisol levels were observed between T1 and T2 (*p* < 0.001, *M*_*Diff*_ = 3.30, 95%-*CI* [1.91, 4.68]) and between T3 and T4 (*p* = 0.003, *M*_*Diff*_= 1.31, 95%-*CI* [0.48, 2.13]), but not between T2 and T3 (*p* = 0.902).

The one-way ANCOVA for the women revealed no main effect of time point (*p* = 0.541).

### Alpha-amylase

One young female, one older female and one older male were excluded from all analysis as outliers.

#### Baseline alpha-amylase

A 2 (time point: T1 vs. T5) × 2 (age group) × 2 (sex) ANCOVA revealed no significant main effects (*p*-values > 0.136). The interaction of time point and sex was significant, *F*_(1, 72)_ = 4.68, *p* = 0.034, η^2^_*p*_ = 0.06. At the start of the session, alpha-amylase levels were descriptively higher for females than males, *p* = 0.067, *M*_*Diff*_ = 32.15, 95%-*CI* [−2.36, 66.65], while there were no significant differences for the samples taken at home, *p* = 0.824, *M*_*Diff*_ = 17.56, 95%-*CI* [−31.08, 38.92]. No other interactions were significant (all *p*-values > 0.123).

#### Alpha-amylase levels throughout the session

A 4 (time point) × 2 (age group) × 2 (sex) mixed ANCOVA revealed a significant main effect of time point, *F*_(2.51, 185.37)_ = 12.65, *p* < 0.001, η^2^_*p*_ = 0.15, alpha-amylase levels increased significantly between time points T1 and T2 (*p* < 0.001, *M*_*Diff*_= −43.75, 95%-*CI* [−56.42, −31.08]), while they remained relatively stable across the remainder of the session (*p*-values > 0.077). Time point interacted with the covariate, *F*_(2.51, 185.37)_ = 6.76, *p* < 0.001, η^2^_*p*_ = 0.08. No other main effects or interactions reached significance (*p*-values > 0.14).

### Positive affect

#### Baseline positive affect

A 2 (time point T1 vs. T5) × 2 (age group) × 2 (sex) mixed ANCOVA revealed a significant main effect of age-group, *F*_(1, 73)_ = 11.16, *p* = 0.001, η^2^_*p*_ = 0.13, indicating that older adults reported higher positive affect. There was a significant three-way interaction, *F*_(1, 73)_ = 5.70, *p* = 0.020, η^2^_*p*_ = 0.07. No other main effects or two-way interactions were significant (all *p*-values > 0.088).

The three-way interaction reflected that young females (*p* = 0.004, *M*_*Diff*_= 0.46, 95%-*CI* [0.15, 0.77]) and older males (*p* < 0.001, *M*_*Diff*_= 0.47 95%-*CI* [0.20, 0.75]) reported significantly higher positive affect during the laboratory session than at home. However, there was no difference for younger male and older female participants (*p*-values > 0.268).

#### Positive affect throughout the session

The 4 (time point) × 2 (age group) × 2 (sex) mixed ANCOVA revealed a significant main effect of age-group, *F*_(1, 77)_ = 25.37, *p* < 0.001, η^2^_*p*_ = 0.25, again showing that older adults consistently reported higher positive affect. This main effect was qualified by a significant interaction of age with time point, *F*_(2.58, 198.57)_ = 12.61, *p* < 0.001, η^2^_*p*_ = 0.14. No other main effects or interactions reached significance (all *p*-values > 0.217).

To follow up on the age × time point interaction, separate one-way ANCOVAs (four levels: T1–T4) were calculated for the young and older adults. Neither for the young adults (all *p*-values > 0.18), nor for the older adults (all *p*-values > 0.57) did the ANCOVA reveal any significant main or interaction effects involving time point. To further investigate the interaction, paired comparisons (T1 vs. T2, T2 vs. T3, T3 vs. T4) were performed. These comparisons revealed no significant change in positive affective rating throughout the session for older adults (*p*-values > 0.366), but a significant decline in positive affect for young adults between T3 and T4 (*p* < 0.001, *M*_*Diff*_= 0.52 95%-*CI* [0.32, 0.72]; all other *p*-values > 0.06).

### Negative affect

Three young males and two older females were excluded as outliers from all analysis of negative affect.

#### Baseline negative affect

A 2 (time point T1 vs. T5) × 2 (age group) × 2 (sex) mixed ANCOVA revealed no significant main or interaction effects (all *p*-values > 0.088).

#### Negative affect throughout the session

A 4 (time point) × 2 (age group) × 2 (sex) mixed ANCOVA revealed no significant main or interaction effects (all *p*-values > 0.15).

### Subjective stress ratings

To determine the subjective stress levels experienced by participants, they were asked to rate their stress on a scale from 0 to 100 after each task. Three separate *t*-tests revealed significant differences after all three tasks. The older adults reported higher stress levels than the younger participants in the episodic memory task, *t*_(80)_ = −2.0, *p* = 0.046, *d* = −0.46, the oddball task, *t*_(73.91)_ = −2.48, *p* = 0.015, *d* = −0.53, and the working memory task, *t*_(76, 54)_ = −4.08, *p* < 0.001, *d* = −0.78.

### Correlation between stress measures and associative memory performance in older adults

Three older adults were excluded from all correlative analysis due to extreme alpha-amylase values at T2 and T3, an extreme negative affective rating at T2 and an extreme negative affective rating at T3, respectively.

**Figure 4:**
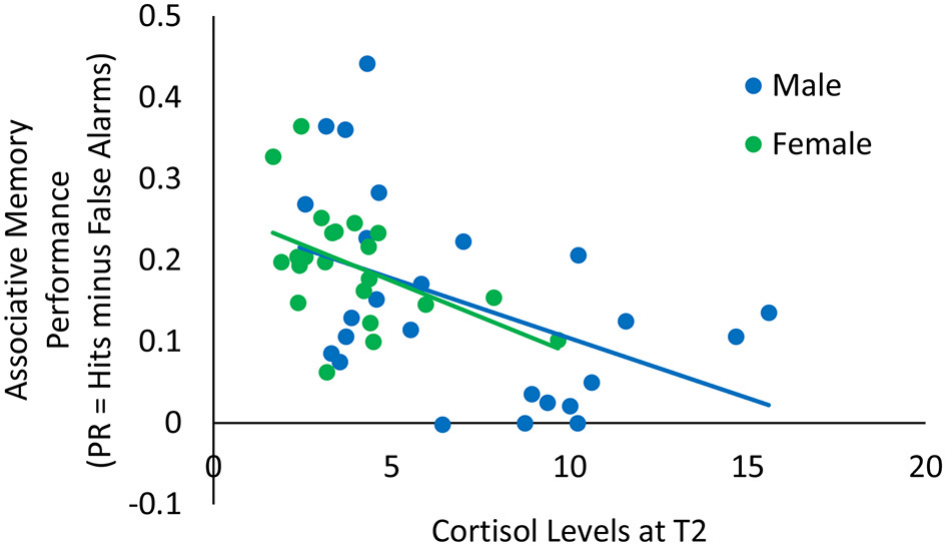
Scatterplot illustrating the relationship between cortisol levels at T2 (*x*-axis) and associative memory performance (*y*-axis) among older males and females.

We examined the correlations between cortisol, alpha-amylase, positive and negative affective ratings before (T2) and after (T3) the memory task with associative memory performance (Table 2). While the correlation between cortisol and associative memory performance is particularly relevant for our hypotheses, we calculated the correlations with all stress/affect measures for completeness. There was a significant negative correlation between cortisol at T2 and associative memory (*r* = −0.50, *p* < 0.001): Lower cortisol levels before the memory task were associated with better associative memory (Figure 4). The negative correlation between cortisol at T3 and associative memory (*r* = −0.31, *p* = 0.029) pointed in the same direction, though it was no longer significant after correction for multiple comparisons. Table 2 presents correlations between each pair of variables.

**Table 2:** Correlation between the stress measures (cortisol, alpha-amylase, positive affect, negative affect) and item and associative memory in older participants (*N* = 49).

**Time point**		**T2**				**T3**			
	**Variable**	**Cortisol**	**Alpha-amylase**	**Positive affect**	**Negative affect**	**Cortisol**	**Alpha-amylase**	**Positive affect**	**Negative affect**
T2	Cortisol								
	Alpha-amylase	−0.04 [−0.32, 0.25]							
	Positive affect	0.24 [−0.05, 0.50]	−0.20 [−0.47, 0.09]						
	Negative affect	−0.02 [−0.31, 0.27]	0.00 [−0.29, 0.29]	−0.10 [−0.38, 0.20]					
T3	Cortisol	**0.67** ^**^ **[0.47, 0.80]**	−0.12 [−0.39, 0.18]	0.18 [−0.12, 0.44]	−0.02 [−0.31, 0.27]				
	Alpha-amylase	0.17 [−0.13, 0.44]	**0.84** ^**^ **[0.73, 0.91]**	−0.14 [−0.41, 0.16]	−0.04 [−0.33, 0.25]	0.12 [−0.18, 0.39]			
	Positive affect	**0.29** ^*^ **[0.00, 0.54]**	−0.10 [−0.38, 0.19]	**0.75** ^**^ **[0.59, 0.86]**	−0.03 [−0.32, 0.26]	0.12 [−0.18, 0.39]	−0.11 [−0.38, 0.19]		
	Negative affect	0.11 [−0.19, 0.39]	0.02 [−0.27, 0.30]	0.01 [−0.28, 0.29]	0.24 [−0.06, 0.49]	0.22 [−0.08, 0.48]	0.07 [−0.22, 0.35]	−0.18 [−0.44, 0.12]	
Associative memory performance		−0.52^**^[−0.70, **−0.27]**	−0.04 [−0.33, 0.25]	0.05 [−0.25, 0.33]	−0.11 [−0.38, 0.19]	−0.30^*^[−0.54, **−0.01]**	−0.19 [−0.46, 0.10]	−0.15 [−0.42, 0.15]	0.08 [−0.22, 0.36]

Values in square brackets indicate the 95% confidence interval for each correlation. ^*^Indicates significance < 0.05, ^**^indicates significance < 0.001. Please note that if corrected for multiple testing all ^**^ are still significant with the largest p = 0.0001. To improve readability, related rows have been highlighted in color. Significant relationships are highlighted in bold to make them easier to identify.

## Discussion

In the present study, we explored affective and physiological responses of young and older adults in a laboratory-based memory experiment, and their association with associative memory performance, while also conducting exploratory analyses of sex differences.

During the laboratory session, but not at home, males showed higher cortisol but lower alpha-amylase levels than females. The sex difference in cortisol within the laboratory was robust across analyses only for the older adults. Cortisol levels tended to decrease across the session for the young adults. Especially for the older females, the relatively low cortisol levels remained stable across the session for the older females, and, by contrast to young adults, the older adults did not show a decline in cortisol from before to after the memory task. The trajectory of alpha-amylase across the experimental session did not differ by age group or sex.

Older adults reported generally more positive affect than young adults, and positive affect further decreased toward the end of the session for young, but not for older adults. For negative affect, we found no significant group effects.

In the older adults, cortisol levels at the time of the memory task were negatively correlated with associative memory performance. While due to sample size restrictions, we did not conduct this analysis separately for males and females, an inspection of Figure 4 suggests that this pattern was similar for both sexes.

Taken together, we found partly sex-specific differences in older vs. young adults in affective responses (cortisol and positive affect) to a laboratory experiment on memory. Furthermore, older adults with higher cortisol levels before the memory task showed lower associative memory performance. In the remainder of the discussion, we will discuss each of our hypotheses individually.

### Are stress markers enhanced in older adults at the beginning of the laboratory session?

We hypothesized that if the laboratory context acts as a stressor, the older participants' salivary cortisol levels would be significantly higher at the start of the session than the younger adults' cortisol levels. Our results show neither differences in cortisol nor alpha-amylase levels between the age groups at the beginning of the session. Additionally, while in males, the cortisol and alpha-amylase levels did not significantly differ from a sample taken at home (although there was a descriptive tendency for higher cortisol in the laboratory in older males), for females, cortisol was lower at the beginning of the session than at home. While conclusions regarding sex differences should be interpreted cautiously, as the subsamples were unbalanced (in the group of young adults) and relatively small, this may suggest that the laboratory context did not act as a stressor in older adults in the sense that it did not lead to enhanced physiological markers of stress at the beginning of the session.

These results contradict (Lupien et al., 2007), who reported that older participants had significantly increased cortisol levels at the beginning of the laboratory session compared to younger adults, and Sindi et al. (2013), who demonstrated significantly higher cortisol levels at the laboratory compared to at home measurements in older adults. However, Lupien et al. (2007) eliminated the difference in cortisol levels by inviting older participants to a group session, where they got to know the laboratory and the experimenters. Additionally, in the experimental session, older participants were accommodated to the environment for 60 min. In our study, the participants were invited to the study over the phone. During the phone call, they were given details on the study procedure, the research topic, and the laboratory. This phone call could have helped to get older participants more familiar with the setting, decreasing nervousness and cortisol levels. This is supported by the finding that there was no age difference in the negative affective rating at the beginning of the session. In fact, the older participants showed a significantly higher positive affective rating at the beginning of the session compared to younger participants. Notably, the negative affective scale includes items such as nervous, anxious, and confused, while the positive affective scale includes items such as interested, excited, and enriched (Krohne et al., 1996). Also, Sindi et al. (2013) could eliminate differences in laboratory vs. at-home cortisol levels by manipulating task instructions. The interaction of a team member with each participant ahead of the experiment could have had a similar effect.

In summary, the present results do not support the idea that the laboratory context of an EEG experiment on memory necessarily acts as a stressor in older adults.

### Does a difficult memory task modulate the expression of stress markers in older adults?

The results of our study highlight age- and sex-specific differences in the cortisol response elicited by a difficult memory task. Younger adults showed a gradual decline in cortisol levels throughout the session. In contrast, exploratory results showed that cortisol level trajectories varied between the older male and female participants: Older males displayed significantly higher cortisol levels across all time points, with a notable decrease only before (from T1 to T2) and after (from T3 to T4) the memory task, but not during it (from T2 to T3). Meanwhile, the relatively low cortisol levels in older females remained stable across the entire session. The older males and older female groups had in common that the decrease in cortisol between T2 and T3, which was observed in young adults, was not observed in older adults. The overall cortisol decrease across the session is a typical pattern and is in accordance with the diurnal cycle of cortisol (Oster et al., 2017). Higher cortisol levels in older males vs. females throughout the session, aligns with the findings of Kudielka et al., 2004, who observed a significantly higher cortisol response to the Trier Social Stress Test (TSST; e.g., Kudielka et al., 2007) in older males compared to females. This suggests that the physiological responses to laboratory conditions and tasks may differ between older males and females. If the difficult memory task itself acts as a stressor, an elevation in cortisol levels from T2 to T3 in older adults would be expected, which was only descriptively observed in older females (Figure 3). This is somewhat consistent with Otte et al. (2005), who noted an increased cortisol response to challenges in older participants, which was more pronounced in women. The challenging memory task in our study may not have been sufficiently stressful to significantly elevate cortisol levels. Nevertheless, it appears to (1) counteract the rapid decline in cortisol levels observed from T1 to T2 in older males and (2) differ from the young adults, whose cortisol levels did decline across the entire session.

To determine the subjective stress levels experienced by participants, they were asked to rate their stress on a scale from 0 to 100 after each task. The findings revealed that older adults generally reported higher stress levels than younger participants, pointing to the complexity of reactions to natural settings. Previous research predominantly focused on external factors like task instructions (Rahhal et al., 2001) or the impact of stressful environments (Lupien et al., 2007; Sindi et al., 2013) on cortisol levels and memory performance across age groups. Our findings suggest that the memory task itself, even if task instructions are worded carefully, can still have a specific effect on the change in cortisol levels in older participants as they do not show a decline in cortisol levels during the memory task. This might be due to the fact that participants already worry about their memory and then are confronted with a memory task that was designed to be relatively difficult in order to avoid ceiling performance. Therefore, the older adults may be confronted with their memory being not as good as they might have hoped. Despite these insights, the lack of significant affective rating changes form before to after the memory task leaves the reasons for age- and sex-specific cortisol level differences unclear. Further studies are needed to assess the role of other hormones, that might interplay with cortisol levels and intraindividual factors such as coping skills or metacognitive beliefs.

### Are physiological stress markers throughout a laboratory-based memory experiment associated with task performance in an associative memory task in older adults?

The correlations between cortisol levels before and after the memory task and associative memory performance were consistently negative for older adults (although after correcting for multiple comparisons only the correlation of cortisol levels before and associative memory performances remained statistically significant). Specifically, lower cortisol levels before and after the memory task were associated with higher associative memory performance. These findings are in line with research suggesting a negative relationship between stress/higher cortisol levels and associative memory performance (Guez et al., 2016; Het et al., 2005).

In an illustrative study (Guez et al., 2016), participants completed an associative memory task both before and after being exposed to TSST. Stress induced by the TSST provoked an “associative deficit” (i.e., a differential impairing effect on associative, but not for item memory) for pictorial stimuli in younger adults.

Although we could not conduct correlational analysis separately for males and females due to sample size restrictions, the pattern was similar for both sexes. Of note, as older males showed higher cortisol levels throughout the session than older females, one might expect females to exhibit higher associative memory performance on the group level. However, no significant differences in the associative memory performance were observed between the sexes [*t*_(50)_ = −1.06, *p* = 0.294]. Our results hence suggest that individual differences within each sex are correlated with memory performance, but third variables that vary between the sexes that may influence cortisol and/or associative memory performance do not necessarily affect these two variables in the same way.

Our results of a similar within-group association of cortisol and associative memory are somewhat at odds with Antypa et al. (2022) and Almela et al. (2011), who found sex-specific differences in the association between memory performance and cortisol-levels. This discrepancy might in part be due to our study not including a single, clearly defined stressful event, but rather an ongoing (potential) stressor before, during and after encoding and retrieval. This makes the interpretation of stressor effects on different phases of the memory cycle more difficult and, together with the absence of an explicit stress manipulation, limits causal conclusions about the observed negative correlation between cortisol and associative memory. Nevertheless, our results serve as a crucial contribution to the field of research by demonstrating that cortisol levels are associated with associative memory performance in older adults, even without a pharmacological intervention or in other explicit stress induction procedure. This highlights the link between physiological/affective responses and cognitive functions in the aging population. Notably, an exploratory analysis in the young adults suggested that the negative association between cortisol levels and associative memory performance may have been unique to our older sample. In the sample of young adults, there was, in fact, a non-significant positive correlation for cortisol at T2 (*r* = 0.31, *p* = 0.091), and a significant positive correlation for cortisol at T3 (*r* = 0.49, *p* = 0.006), with associative memory performance. Given that our younger sample was small, it is important to view these correlations with caution, and for future research to include a larger younger group of participants. This expansion will enable a thorough investigation to determine if these correlations present similarly across age groups, ensuring that observations like the association deficit are unilaterally influenced by age-specific factors.

### Limitations and general conclusion

The present research question was examined within a typical experiment on adult age differences in memory function. Although participants followed the typical instructions for studies examining psychosocial stress (such as regarding eating and drinking before the session), other factors e.g., the time of the day during with the session was completed were varied between participants. We statistically controlled for the time of the session and, in line with the well-described diurnal changes in cortisol and other physiological measures (Elverson and Wilson, 2005), the covariate was consistently predictive of cortisol levels. However, it should be noted that non-linear effects such as a reduced cortisol response to the task in the morning due to already elevated baseline levels cannot be fully accounted for by statistical control using ANCOVA.

An additional limitation is that the second and third saliva samples, which were the most relevant for the present study, were spaced nearly an hour apart. Thus, we did not capture the precise temporal dynamics of the cortisol response throughout the memory task and cannot dissociate between effects on encoding, consolidation and retrieval.

Future studies should hence collect a sufficiently large sample of participants such that the time of the session could be analyzed as an experimental factor rather than a statistical covariate and incorporate more frequent saliva sampling to improve the temporal resolution of physiological response measurement. Moreover, due to the primary research question of the study not including sex differences, the subsamples of young males and females were not equally large. This should be balanced out in future studies.

It should also be noted that several factors besides age, sex and time of day can influence cortisol levels. We addressed this issue in multiple ways. First, acute psychiatric disorders were an exclusion for study participation, as such conditions can alter cortisol levels in complex ways (for a review, see George et al., 2025). We further recorded whether participants had a history of neurological or psychiatric care. The four groups (age × sex) did not differ significantly in this aspect [χ^2^_(3)_ = 5.45, *p* = 0.142, ϕ = 0.26].

Medication is another important source of variance. Hormonal contraceptives in particular are known to affect cortisol trajectories (Gervasio et al., 2022). In our sample nine out of 19 young women used hormonal contraceptives. When excluding these participants from the cortisol trajectory analyses, the overall pattern of results remained unchanged. Beyond contraceptives, five younger participants (one taking an antihistamine, one probiotics and three thyroid medication) and 36 older adults reported current medication (e.g., blood thinners, antihypertensives, antidiabetics, cholesterol-lowering agents, antihistamines, thyroid medication). There are studies reporting the influence of specific medications on cortisol (Granger et al., 2009), and the effects of these medications on cortisol can be heterogeneous and may act in opposing directions. Given the heterogeneity of medication within the older group, we refrained from including medication as a covariate, which represents a limitation of the present study.

Another important factor influencing cortisol levels is smoking (Badrick et al., 2007). In our sample, five younger and four older participants smoked, which did not significantly differ between the groups [χ^2^_(3)_ = 3.38, *p* = 0.337, ϕ = 0.20].

Finally, there remain factors that are inherently difficult to control, such as the profound hormonal differences between younger and older women. Nevertheless, after carefully considering and testing several potential confounders, we believe that the current findings provide a valuable contribution to understanding cortisol patterns in younger and older adults in the context of a laboratory experiment.

In conclusion, while the laboratory context did not induce higher stress levels at the beginning of the session for older participants, our study revealed distinct age- and sex-specific differences in how the laboratory session including a difficult memory task influenced stress markers and affective responses, and in turn, memory performance. This suggests that the experimental tasks conducted in the laboratory may in some circumstances modulate affective responses, particularly for older individuals. It may hence be advisable in some circumstances to capture physiological and psychological measures of affect during experiments on age differences in cognitive processes.

Perhaps most importantly, cortisol levels showed a robust negative correlation with associative memory performance in older adults. Physiological factors may hence contribute to the typically pronounced age differences in (associative) episodic memory, and such contributions should be examined further to better understand the mechanisms behind the associative memory deficit in older adults.

## Data Availability

The raw data supporting the conclusions of this article will be made available by the authors, without undue reservation.

## Footnotes

^1^A reviewer suggested to additionally include baseline cortisol (cortisol levels at home) as a covariate. A control analysis in which we followed this suggestion, revealed analogous results to those reported in this article.
