# Supplementary material for: An intricate relationship: stress markers and associative memory in a laboratory experiment in older adults
Source: Front Aging Neurosci. 2025 Oct 29;17:1666566. doi: 10.3389/fnagi.2025.1666566 (PMC12605387; doi:10.3389/fnagi.2025.1666566)
Supplement: Supplementary file 1 [file Data_Sheet_1.pdf]

Statistical analysis

To address the issue that the cortisol and amylase data were not normally distributed, we repeated our statistical analysis with log-transformed(ln) data. To ensure comparability, the same outliers as before were excluded before data transformation. After the transformation, the distribution of the data did not deviate from a normal distribution, as indicated by a non-significant Kolmogorov-Smirnov-Test. We then proceeded with the same statistical analysis approach as described in the article:

We statistically analyzed the dependent variables in mixed ANCOVAs with the within-subject factor time point and the between subject factors age group (young vs. old) and sex (male vs. female), using IBM SPSS 28 software. Greenhouse-Geisser correction was applied where necessary. The start time of the experimental session was included in all ANCOVAs as a covariate to account for the diurnal cycle of cortisol. We report partial eta squared (η^2_p) as a measure of effect size. Significant main effects of factors with more than one level and interactions were followed by lower level ANCOVAs or Fishers LSD.

Results

Cortisol

One young female was excluded from all analysis of cortisol as an outlier.

Baseline Cortisol

A 2 (time point: T1 vs. T5) x 2 (age group) x 2 (sex) mixed ANCOVA revealed a significant effect of the covariate, $F(1, 73) = 85.21$, $p < .001$, $\eta^2_p = .54$, and a significant interaction of time point and sex, $F(1, 73) = 4.49$, $p = .037$, $\eta^2_p = .06$. For males, cortisol levels were significantly higher at the start of the session than for females, $p = .014$, $M_{Diff} = .31$, 95%-CI[.06, .55], while there were no significant sex differences for the samples taken at home, $p = .944$, $M_{Diff} = -.01$, 95%-CI[-.28, .26]. This was driven by the fact that females had a lower cortisol level at the beginning of the session than at home, $p = .034$, $M_{Diff} = -.21$, 95%-CI[-.41, -.02], while males did not differ in their cortisol levels between the session and at home, $p > .361$. Furthermore, a significant age group x sex interaction, $F(1, 73) = 6.47$, $p = .013$, $\eta^2_p = .08$, revealed that, overall, older males had significantly higher cortisol levels than older females, $p = .002$, $M_{Diff} = .42$, 95%-CI[.16, .67], while there was no significant

difference between younger males and females, p = .482, M_{Diff} = -.12, 95%-CI[-.46, .22]. There were no other main or interaction effects (all p-values > .43).

The results for the baseline cortisol were hence analogous to the analyses on the non-transformed data.

Cortisol levels throughout the session

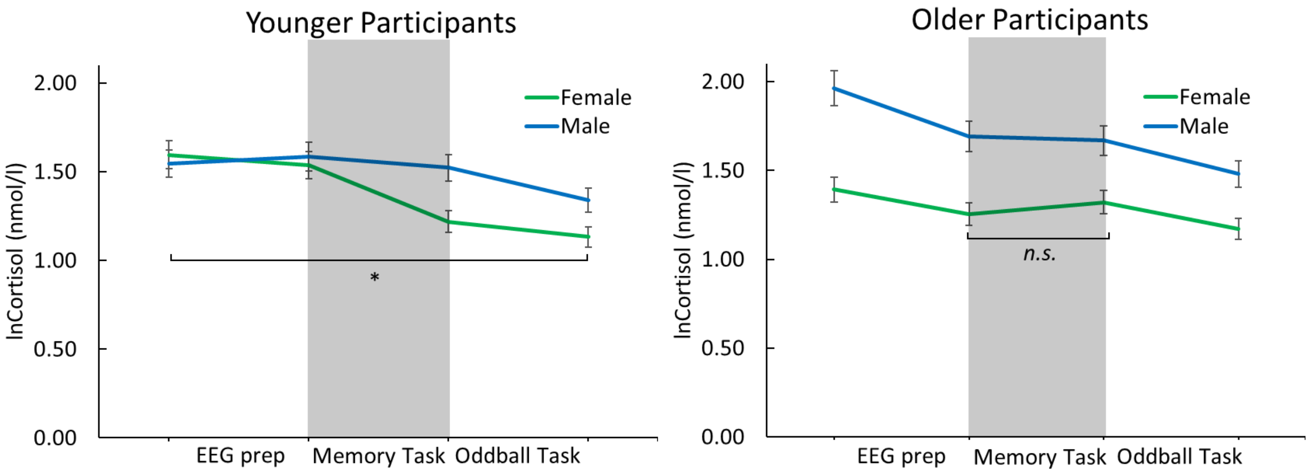

Figure S1. Estimated mean cortisol levels with consideration of the covariate start time of older and younger males and females throughout the session. The error bars indicate the 95% confidence interval. *indicates significance <.05.

A 4 (time point: T1-T4) x 2 (age group) x 2 (sex) mixed ANCOVA, revealed a significant effect of the covariate, $F(1, 76) = 56.84$, $p < .001$, $\eta^2_p = .43$, and of sex, $F(1, 76) = 7.47$, $p = .008$, $\eta^2_p = .09$, such that males had higher cortisol levels than females. There was a main effect of time point, $F(1.59, 121.06) = 6.24$, $p = .005$, $\eta^2_p = .08$, and an interaction of time point with the covariate, $F(1.59, 121.06) = 3.36$, $p = .048$, $\eta^2_p = .04$. Unlike in the main analysis of raw cortisol values, the three-way interaction of time point, age group, and sex did not reach significance, $F(1.59, 121.06) = 2.19$, $p = .127$. However, there were significant interactions between age group and the cubic trend of time point, $F(1, 76) = 4.17$, $p = .045$, $\eta^2_p =.052$, as well as between age group, sex and the quadratic trend of time point, $F(1, 76) = 4.24$, $p = .043$, $\eta^2_p = .05$, suggesting that the time course of cortisol levels over the session took a different shape in young vs. older adults (which was in part modulated by sex; Figure S1). To follow up on these interactions, separate 4 (time point) x 2 (sex) ANCOVAs were calculated for the young and older adults.

Young adults

For the young adults, there was a significant effect of the covariate, $F(1, 26) = 27.58, p < .001$, $\eta^2_p = .57$, and of time point, $F(1.60, 41.68) = 5.60, p = .011$, $\eta^2_p = .18$. Regarding the main effect of time point only the linear trend was significant, $F(1, 26) = 6.91, p = .014$, $\eta^2_p = .21$. There was no main or interaction effect involving the factor sex.

Older adults

For the older adults, aside from an effect of the covariate, $F(1, 49) = 25.68, p < .001$, $\eta^2_p = .34$, there was a main effect of sex, $F(1, 49) = 11.79, p =.001$, $\eta^2_p = .19$, with higher cortisol levels in male participants. The main effect of time point did not reach significance, $F(1.51, 74.12) = 2.60, p = .095$, $\eta^2_p = .05$. Indicating that in contrast to the younger participants, the decrease in cortisol levels throughout the session was not significant No other effects reached significance in the ANOVA. Notably, a targeted analysis on timepoints T2 and T3, collapsed across sexes (but including the covariate), revealed that the mean cortisol level was almost identical (T2: M=1.56, SD=.59; T3: M= 1.56, SD = .64) and the difference was not significant, $F(1, 50) = .18, p =. 677$.

Taken together, the result pattern of the statistical analysis of the transformed cortisol data, despite some minor differences, supported the conclusion of our main analysis, that between time point T2 and T3, where the memory task was completed, cortisol levels decreased for the young, but not for the older adults.

Alpha-Amylase

One young female, one older female and one older male were excluded from all analysis as outliers.

Baseline Alpha-Amylase

A 2 (time point: T1 vs. T5) x 2 (age group) x 2 (sex) ANCOVA revealed no significant main effects (p-values > .273). The interaction of time point and sex was significant, $F(1, 72) = 7.45, p = .008$, $\eta^2_p = .09$, as well as the three-way interaction of time point, sex and age group. For young males, alpha-amylase levels were higher at the start of the session than at home, $p = .007$, $M_{Diff} = .43$, 95%-*CI*[.12, .74], while there were no significant differences for young females, $p = .106$, $M_{Diff} = -.20$, 95%-

CI[-.44, .04], for older females, $p = .500$, $M_{Diff} = -.07$, 95%-*CI*[-.14, .29] and for older males, $p = .760$, $M_{Diff} = -.03$, 95%-*CI*[-.24, .18]. No other interactions were significant (all *p*-values > .187).

The results of baseline vs. laboratory amylase levels was hence largely analogous to the main analyses on the raw data, except that with the transformed data, the difference between the samples taken at home and those taken in the lab was significant only for the young males (as opposed to males regardless of age).

Alpha-Amylase levels throughout the session

A 4 (time point) x 2 (age group) x 2 (sex) mixed ANCOVA revealed a significant main effect of time point, $F(2.34, 172.87) = 20.01$, $p < .001$, $\eta^2_p = .21$. Time point interacted with the covariate, $F(2.34, 172.87) = 7.86$, $p < .001$, $\eta^2_p = .10$. A two-way interaction of time point and sex could also be observed, $F(2.34, 172.87) = 2.97$, $p = .046$, $\eta^2_p = .04$. For males there was a significant increase of alpha-amylase levels between T1 and T2, $p < .001$, $M_{Diff} = .42$, 95%-*CI*[.28, .56], and between T2 and T3, $p = .020$, $M_{Diff} = .13$, 95%-*CI*[.02, .23], and no significant increase between T3 and T4, $p = .235$, $M_{Diff} = .05$, 95%-*CI*[-.03, .13]. For females there was only a significant increase of alpha-amylase levels between T1 and T2, $p < .001$, $M_{Diff} = .33$, 95%-*CI*[.21, .45] (other p-values > .559). No other main effects or interactions reached significance (*p*-values > .100).
