# Supplementary material for: An intricate relationship: stress markers and associative memory in a laboratory experiment in older adults
Source: Front Aging Neurosci. 2025 Oct 29;17:1666566. doi: 10.3389/fnagi.2025.1666566 (PMC12605387; doi:10.3389/fnagi.2025.1666566)
Supplement: Supplementary file 2 [file Data_Sheet_2.pdf]

General Instructions:

Welcome to this task! This task consists of several parts.

1) First, you will complete two learning tasks in two sections.
2) The learning tasks will be followed by a separate counting task.
3) Finally, two memory tests will follow, each corresponding to one of the two learning tasks from the first part.

Item Task Instructions:

In the first learning task, you will judge whether objects can be eaten or drunk, or not.

It is important that you do not judge based on whether you personally like to eat the given object, but rather on whether it belongs to human food. Furthermore, you should judge the object as it is presented. For example, if you are shown a cow, you should classify it as "not edible," even though in processed form livestock are often part of human food.

Please press the [EdibleButton] for edible objects and the [InedibleButton] for inedible objects. This key assignment will also be displayed at the bottom of the screen during the task. Please respond to each object as quickly as possible by pressing the corresponding key.

After some time, a memory test will follow for the objects from this learning task.
In the memory test, you will be shown objects from the learning task as well as objects that did not appear.

Your task is to judge whether a given object is "old," meaning it was shown during the learning phase, or "new," meaning it was not shown during the learning phase.

You will give your judgment of whether an object is "old" or "new" on a scale from 1 to 6.
Press 1 if you clearly know that the object is definitely old. Press 6 if you clearly know that the object is definitely new. Keys 2–4 represent different degrees of certainty about your judgment.

While you should respond as quickly as possible in the learning task, you may take more time to respond in the memory test. The object will remain on the screen until you have given your response.

Association Task Instructions:

You will now get to know the second learning task.

In the second learning task, you will judge for PAIRS of objects whether one object fits into the other.

You should imagine the objects in their real-life size and not judge according to the size of the pictures on the screen. Furthermore, it is important that you base your judgment on the objects as they are presented. You may mentally rotate the objects, but you may not change their shape.

Please press the [FitButton] if one object fits into the other, and the [NoFitButton] if this is not the case. This key assignment will also be displayed at the bottom of the screen during the task. Please respond to each object pair as quickly as possible by pressing the corresponding key.

After some time, a memory test will follow for the objects from this second learning task.

In the memory test, you will be shown pairs from the learning task that were presented in exactly this combination. In addition, you will be shown pairs that consist of objects from two different learning pairs. In the latter case, the combination did not occur. It is important to emphasize that NO ENTIRELY NEW objects will appear; only the COMBINATION of two stimuli can be new (or not).

Your task is to judge whether a given object pair is "old," meaning it was shown in exactly this combination during the learning phase, or "new," meaning it was not shown in this combination during the learning phase (even if each of the objects did appear during the learning phase as part of another pair).

As in the first memory test, you will give your judgment of whether an object pair is "old" or "new" on a scale from 1 to 6.
Press 1 if you clearly know that the object pair is definitely old. Press 6 if you clearly know that the object pair is definitely new. Keys 2–4 represent different degrees of certainty about your judgment.

While you should respond as quickly as possible in the learning task, you may take more time to respond in the memory test. The object will remain on the screen until you have given your response.
